# Clostridioides difficile Infection in Hematology-Oncology Patients Receiving Bacterial Prophylaxis With Levofloxacin: A Single-Institution Experience

**DOI:** 10.7759/cureus.105973

**Published:** 2026-03-27

**Authors:** Aleksandra M Sobiborowicz-Sadowska, Emilie Chebat, Dennis J Kuo

**Affiliations:** 1 Department of Pediatrics, UC San Diego Health, San Diego, USA; 2 Pharmacology, Rady Children's Hospital, San Diego, USA; 3 Division of Pediatric Hematology-Oncology, UC San Diego Health, San Diego, USA

**Keywords:** clostridioides difficile infection, diarrhea, levofloxacin, neutropenia, prophylaxis

## Abstract

Background and objectives

Prophylactic levofloxacin reduces the risk of bacteremia in patients with treatment-induced neutropenia. However, fluoroquinolone use has been linked to an increased risk of Clostridioides difficile infections (CDI), which can be associated with delays in treatment schedules or prolonged hospital stays in hematology/oncology patients. Due to a lack of convincing evidence, routine CDI prophylactic interventions are not currently recommended. In this context, we aimed to assess how prophylactic levofloxacin in pediatric hematology-oncology patients affects CDI rates at our institution and whether there is a rationale for implementing CDI prophylactic measures in this population.

Methods

We conducted a retrospective chart review of prophylactic levofloxacin administration records in patients admitted to our pediatric hematology-oncology unit between January 2022 and June 2023. Only records documenting more than four days of continuous exposure to levofloxacin were included. Patients were excluded if levofloxacin was administered exclusively in the outpatient setting, if they did not have a primary hematologic or oncologic diagnosis, or if levofloxacin was administered for purposes other than antibacterial prophylaxis. Cases of CDI were identified as the presence of diarrhea with a positive stool C. difficile toxin A/B enzyme immunoassay or nucleic acid amplification test. CDIs that occurred within 30 days of documented levofloxacin exposure were then reported.

Results

We found 408 records of levofloxacin exposure in 234 patients between January 2022 and June 2023. Seventy-five patients included in the final analysis received a total of 1851 days of levofloxacin prophylaxis in 129 recorded exposures (mean: 14.35 days per exposure, range: 4-76 days). CDI occurred in 10 patients (13.3%) at some point after a hematologic or oncologic diagnosis had been established. We identified five cases of CDI within 30 days of the last day of levofloxacin prophylaxis; thus, CDI occurred in 6.67% of all patients and 3.88% of all exposures. Three of these cases occurred after cessation of levofloxacin and the administration of intravenous broad-spectrum antibiotics for febrile neutropenia. Only two of the patients who developed CDI within that time frame had not received any antibiotics other than levofloxacin immediately before the infection. One of these patients had complete resolution of the infection with an oral antibiotic course, while the other died within six days due to unrelated infectious causes.

Conclusions

Levofloxacin antimicrobial prophylaxis in neutropenic patients does not appear to be associated with an increased risk of CDI or with an increased risk of adverse outcomes from the infection. This study does not provide evidence supporting the need for additional CDI prophylactic measures, such as antibiotics, in these patients.

## Introduction

Bacterial infections in pediatric patients with treatment-induced neutropenia represent a major cause of hospitalization and mortality, constituting a significant burden to patients and their providers [1]. Prophylactic administration of fluoroquinolone antibiotics, such as levofloxacin, in treatment-induced neutropenia has been shown to reduce the risk of bacteremia in both adult [2] and pediatric [3,4] cohorts. Accordingly, fluoroquinolone antibiotics have been recommended by the Infectious Diseases Society of America for the prophylaxis of bacterial infections in high-risk neutropenic patients [5]. Thus, levofloxacin prophylaxis is now incorporated as standard practice for management of such neutropenic patients at our institution, and involves administration of levofloxacin to hematology/oncology patients with neutropenia (defined as absolute neutrophil count <500/µl) at a dose of 10 mg/kg BID (max 250 mg/dose, or 500 mg/day) for patients aged ≥6 months to <5 years or 10 mg/kg once daily (max 750 mg/day) for patients aged ≥5 years, who are currently not receiving other antibiotics for an active infection. 

However, fluoroquinolone use has previously been linked to an increased risk of developing Clostridioides difficile infection (CDI) [6]. In pediatric cancer patients, diarrhea caused by CDI is associated with events such as delays in treatment schedules or prolonged hospitalizations [7]. Due to a lack of convincing evidence, neither the clinical practice guideline developed by the Pediatric Oncology Group of Ontario [8] nor that developed by the American College of Gastroenterology [9] currently recommends routine CDI prophylactic interventions. However, given the significant burden of care associated with CDI, there is interest in using prophylactic measures such as oral vancomycin administration to prevent CDI in pediatric cancer patients [10]. 

As pediatric hematology-oncology patients with treatment-induced neutropenia frequently receive prolonged courses of prophylactic levofloxacin, there is concern that this practice could be associated with increased rates of CDI. Thus, in this study, our goal was to assess the rate of CDI among hematology-oncology patients receiving prophylactic levofloxacin during periods of neutropenia at our institution and to evaluate whether these findings support the need for additional CDI prophylactic measures in this population.

## Materials and methods

We conducted a retrospective chart review that was approved by the Institutional Review Board of the University of California, San Diego. The criterion for the initial search was inpatient administration of levofloxacin recorded between January 2022 and June 2023. The inclusion criteria were as follows: patients admitted to the Hematology/Oncology Division; those treated for a primary hematologic or oncologic diagnosis (malignancy or a chronic benign hematologic diagnosis such as aplastic anemia, congenital neutropenia, leukocyte adhesion deficiency, or MDS); patients with neutropenia associated with the primary diagnosis or with treatment; and those in whom levofloxacin was administered in the inpatient setting as bacterial prophylaxis. Patients were excluded if levofloxacin was administered exclusively in the outpatient setting, if the patient did not carry a primary hematologic or oncologic diagnosis, or if levofloxacin was administered for purposes other than antibacterial prophylaxis.

We defined each period of continuous administration of levofloxacin prophylaxis as a single recorded exposure. Sequential exposures to levofloxacin with fewer than five days between them were considered a single recorded exposure unless they were separated by the administration of other broad-spectrum antibiotics. Additionally, recorded exposures of fewer than four days in duration were excluded from the study, as shorter courses have not been associated with an increased risk of CDI in the literature [11].

The included patients' records were manually reviewed by the authors to document the reason for cessation of levofloxacin prophylaxis (e.g., discharge home, recovery of neutrophil counts, or administration of intravenous antibiotics for febrile neutropenia) and to identify instances of CDI within our cohort. The primary outcome was the incidence of CDI in our cohort. CDI was defined as the presence of clinical symptoms (diarrhea) in the setting of a positive stool C. diff toxin A/B enzyme immunoassay or nucleic acid amplification test (NAAT). Records of patients who developed CDI within 30 days of documented levofloxacin exposure were further analyzed for secondary outcomes (CDI resolution, defined as clinical resolution of diarrhea, versus development of an adverse event, defined as toxic megacolon, intestinal perforation, admission to the pediatric ICU, or death). The analysis of the outcome data was limited to descriptive measures due to the low number of CDI cases identified.

## Results

**Figure 1:**
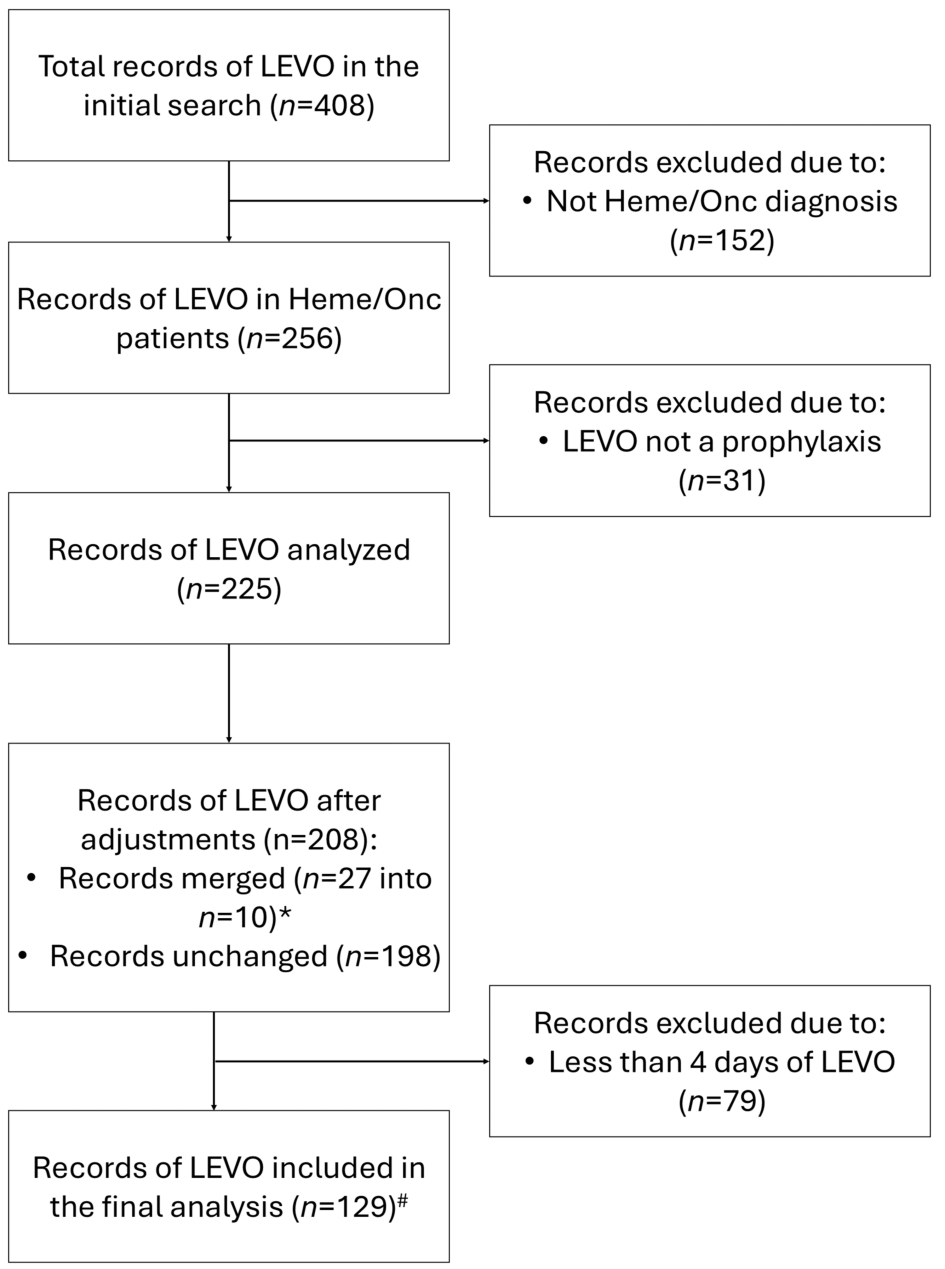
Flowchart of records of levofloxacin (LEVO) exposure identified in the primary search and included in the final analysis ^*^Sequential exposures to levofloxacin within less than 5 days were merged into one continuous recorded exposure unless separated by administration of other antibiotics. ^#^The final analysis included 129 records of levofloxacin exposure in 75 patients (whose demographic characteristics were presented in Table 1), as 28 patients had multiple separate exposures documented Data presented as number of records, n

Between January 2022 and June 2023, we found 408 records of levofloxacin exposure, as presented in Figure 1. After the removal of 183 records that met the exclusion criteria (n=152 - due to primary diagnosis other hematologic/oncologic, and n=31 - due to levofloxacin given not as prophylaxis), the remaining 225 records were analyzed and adjusted as described above. Subsequently, 79 records of prophylactic levofloxacin exposure of less than four days were identified and excluded. Thus, the final analysis included 129 records of levofloxacin exposure in 75 patients, as 28 patients had multiple separate exposures documented. The demographic characteristics and primary diagnoses of the included patients are presented in Table 1.

**Table 1:** Demographic characteristics and primary diagnoses of the study population (75 patients whose levofloxacin exposures were included in the final analysis)

Demographic characteristics		
Age at levofloxacin exposure, years, mean (range)		11.08 (0.34-20)
Sex, n (%)		
	Male	40 (53.33)
	Female	35 (46.67)
Primary diagnosis, n (%)		
	Acute lymphoblastic leukemia	45 (60)
	Acute myeloblastic leukemia	10 (13.33)
	Aplastic anemia	5 (6.67)
	Non-Hodgkin lymphoma	4 (5.33)
	Myelodysplastic syndrome	2 (2.67)
	Mixed lineage leukemia	2 (2.67)
	Chronic myelogenous leukemia	1 (1.33)
	Hodgkin lymphoma	1 (1.33)
	Congenital neutropenia	1 (1.33)
	Medulloblastoma	1 (1.33)
	Neuroblastoma	1 (1.33)
	Atypical teratoid rhabdoid tumor	1 (1.33)
	Leukocyte adhesion deficiency	1 (1.33)

Seventy-five patients included in the final analysis received a total of 1851 days of levofloxacin prophylaxis in 129 recorded exposures (median: 11 days per exposure, range: 4-76 days). In our cohort, CDI occurred in 10 patients (13.3%) at some point after a hematologic/oncologic diagnosis was made. In five of those, CDI occurred within 30 days of the last day of levofloxacin prophylaxis (Table 2). Thus, shortly after levofloxacin exposure, CDI occurred in 6.67% of patients and 3.88% of exposures. Of note, three patients developed CDI after completing levofloxacin prophylaxis and receiving intravenous broad-spectrum antibiotics for febrile neutropenia. All three patients achieved full resolution of the infection following treatment with oral antibiotics targeted against CDI. Two patients developed CDI within 30 days of levofloxacin exposure without having received additional antibiotics immediately before the infection. One of these patients experienced full resolution with oral antibiotics, while the other died due to unrelated infectious causes. Four of the five identified cases were considered primary, as only one patient had a prior recorded infection in their medical record.

**Table 2:** Characteristics of patients with documented CDI within 30 days of prophylactic levofloxacin CDI: Clostridioides difficile infection; AA: aplastic anemia; ALL: acute lymphoblastic leukemia; AML: acute myeloblastic leukemia; NAAT: nucleic acid amplification test; ppx: prophylaxis; LEVO: levofloxacin; toxin: toxin A/B enzyme immunoassay

Age (years), sex, primary diagnosis	LEVO ppx days (N)	Antibiotics after LEVO	CDI criteria	Primary infection	Clinical outcome
18, male, ALL	21	No	Diarrhea and toxin (+)	Yes	Expired within 6 days due to unrelated causes
7, male, AML	26	No	Diarrhea and toxin (+) and NAAT (+)	Yes	Resolved with oral antibiotics
4, female, AA	8	Yes	Diarrhea and toxin (+) and NAAT (+) and pseudomembranous colitis in colonoscopy	Yes	Resolved with oral antibiotics
14, male, ALL	14	Yes	Diarrhea and NAAT (+)	No	Resolved with oral antibiotics
10, female, ALL	6	Yes	Diarrhea and toxin (+) & NAAT (+)	Yes	Resolved with oral antibiotics

## Discussion

Pediatric cancer patients represent the second most common group among pediatric patients with CDI-associated medical encounters, following children with chronic gastrointestinal conditions [12]. Consequently, CDI rates in pediatric cancer populations remain high, with reported prevalence ranging from 11.4% [7] to 38.2% [13] of all patients. In this retrospective study, we found a similar overall prevalence of CDI in hematology and oncology patients, reaching 13.33% of our analyzed cohort. Several risk factors that predispose patients to CDI are highly prevalent in this population, including prolonged hospitalizations, immunodeficiency related to both the primary diagnosis and its treatment, and frequent exposure to a variety of broad-spectrum antibiotics [14,15].

Importantly, the incidence of CDI among patients receiving levofloxacin prophylaxis in our study was low, with 6.67% developing CDI within 30 days of the last day of exposure. Additionally, three of these five patients had received other broad-spectrum antibiotics for febrile neutropenia before CDI, making it uncertain whether the infection was directly related to prophylactic levofloxacin exposure. Overall, our study did not find evidence that fluoroquinolone prophylaxis in this population of pediatric hematology and oncology patients was associated with a significantly increased risk of CDI. This supports the limited data currently available in the literature regarding CDI in pediatric hematology-oncology patients.

In a multicenter, open-label, randomized trial of levofloxacin prophylaxis in a cohort of pediatric patients with acute lymphoblastic leukemia (ALL), no differences in CDI rates were observed between patients who did or did not receive levofloxacin [3]. Additionally, a retrospective chart review of pediatric and adult patients undergoing stem cell transplantation showed that levofloxacin prophylaxis was associated with a lower risk of CDI compared with no prophylaxis, which the authors attributed to a reduced rate of bacterial infections and decreased overall antibiotic exposure in patients receiving levofloxacin [4]. It is important to note that the cohort not receiving levofloxacin prophylaxis consisted of patients admitted before the drug was incorporated into institutional practice, increasing the potential for confounding factors to influence these findings.

CDI in the oncology population has been shown to negatively impact clinical outcomes, including prolonged hospitalizations, delayed administration of oncologic treatments [7], and severe adverse events requiring intensive care such as vasopressor support or mechanical ventilation [16]. In our study, patients who developed CDI shortly after levofloxacin prophylaxis achieved complete resolution of symptoms with first-line oral antibiotic treatment, except for one patient who died due to unrelated causes. Therefore, our findings suggest that prophylactic levofloxacin is not associated with a significantly increased risk of CDI-related adverse events.

Well-established CDI preventive measures, including patient isolation, hand hygiene, and the use of contact precautions, are highly effective in reducing the incidence of CDI in clinical practice [17]. Based on our findings, there is little justification for implementing additional or novel CDI preventive measures in patients receiving levofloxacin prophylaxis, particularly since such interventions can carry their own risks. For example, oral vancomycin used for primary CDI prophylaxis has been reported to increase the risk of gram-negative bacteremia in patients undergoing stem cell transplantation [18]. Overall, our findings support the recommendations of the clinical practice guidelines from the Pediatric Oncology Group of Ontario and the American College of Gastroenterology [8,9], as there is likely no indication for additional CDI prevention in this context. However, given the high CDI recurrence rates observed in the pediatric oncology population [19] and the elevated rates of treatment failure in children with recurrent CDI [20], the use of secondary CDI prevention measures in patients with a history of C. difficile-associated diarrhea is more justifiable [21]. 

We acknowledge several limitations of this study. It was based on a retrospective chart review, included a relatively small sample size, and observed a low incidence of C. difficile infections. Consequently, our data analysis was limited to descriptive measures, which nevertheless provide insights into this adverse event in pediatric cancer patients receiving levofloxacin for bacterial prophylaxis, reflecting real-world clinical practice. This study included both hematology and oncology patients, as they share several clinically relevant characteristics central to the study question, namely neutropenia, immunocompromised status, and the prophylactic use of levofloxacin. Additionally, hematology and oncology patients are often hospitalized on the same inpatient units and managed by the same clinical staff, resulting in comparable levels of exposure to C. difficile. However, this is a heterogeneous population with respect to underlying diagnoses, chemotherapy regimens, and prior antibiotic exposure, which is an important consideration when interpreting our findings.

## Conclusions

CDIs are commonly associated with broad-spectrum antibiotic use and are prevalent in the pediatric hematology and oncology population. In our study population of neutropenic patients admitted to the hematology-oncology service, levofloxacin prophylaxis was not associated with an increased risk of CDI or with worse outcomes from CDI. Therefore, our study does not provide evidence supporting the need for additional CDI prophylactic measures, such as antibiotics, in these patients. However, the generalizability of our findings is limited by the retrospective design, small sample size, and heterogeneous patient population. Given that most currently available evidence is derived from retrospective studies subject to confounding, further long-term prospective surveillance of neutropenic patients receiving antibacterial prophylaxis is warranted.
